# *Drosophila* UTX Coordinates with p53 to Regulate *ku80* Expression in Response to DNA Damage

**DOI:** 10.1371/journal.pone.0078652

**Published:** 2013-11-12

**Authors:** Chengwan Zhang, Zehui Hong, Wencui Ma, Da Ma, Yuchen Qian, Wei Xie, Feng Tie, Ming Fang

**Affiliations:** 1 Institute of Life Sciences, Southeast University, State Ministry of Education Key Laboratory of Developmental Genes and Human Diseases, Nanjing, China; 2 Department of Genetics and Developmental Biology, Medical School of Southeast University, Nanjing, China; 3 Department of Genetics and Genome Sciences, Case Western Reserve University, Cleveland, Ohio, United States of America; Philipps University, Germany

## Abstract

UTX is known as a general factor that activates gene transcription during development. Here, we demonstrate an additional essential role of UTX in the DNA damage response, in which it upregulates the expression of *ku80* in *Drosophila*, both in cultured cells and in third instar larvae. We further showed that UTX mediates the expression of *ku80* by the demethylation of H3K27me3 at the *ku80* promoter upon exposure to ionizing radiation (IR) in a p53-dependent manner. UTX interacts physically with p53, and both UTX and p53 are recruited to the *ku80* promoter following IR exposure in an interdependent manner. In contrast, the loss of *utx* has little impact on the expression of *ku70*, *mre11*, *hid* and *reaper*, suggesting the specific regulation of *ku80* expression by UTX. Thus, our findings further elucidate the molecular function of UTX.

## Introduction

Maintaining genomic stability is crucial for ensuring the accurate cellular functioning of organisms ranging from bacteria to humans [1], [2]. During the course of evolution, cells have evolved multiple mechanisms, collectively known as the DNA damage response (DDR), that facilitate the cellular response to DNA damage [3], [4], [5]. These mechanisms include cell-cycle arrest, DNA repair and apoptosis [6], [7]. In addition, cells responding to DNA damage display a specific gene expression profile that facilitates DNA repair [8]. For example, CSA and HR23A are upregulated by the transcription factor USF-1 in response to UV damage [9]. In the normal diploid human lung fibroblast line MRC-5, exposure to ionizing radiation results in the upregulation of Ku70 via a p53/ATM-dependent mechanism [10]. DNA damage induces *CRT1* transcription, which is downstream of *DUN1* in the DNA damage pathway in yeast. In turn, *CRT1* becomes hyperphosphorylated and dissociates from DNA, resulting in the transcriptional induction of three of the four *RNR* genes [11]. Over the last few years, a wealth of new information has been uncovered about the DDR, including the identification of many novel proteins involved in this process [12], but whether these proteins are regulated at the gene transcription level in response to DNA damage remains poorly understood.

The *ubiquitously transcribed TPR gene on the X chromosome*, or *UTX*, was first described as a gene that escapes from X chromosome inactivation [13], [14]. It is now clear that the *UTX* gene encodes a JmjC-domain-containing protein with histone lysine demethylase activity specific for the tri-methylated lysine 27 residues of histone H3 (H3K27me3) [15], [16], [17], [18], [19], [20], and it is officially referred to as *KDM6A* in the human genome. Several recent studies have found that UTX is a major component of the COMPASS complex, which includes myeloid/lymphoid or mixed-lineage leukemia (MLL), a SET-domain containing protein homologous to *Drosophila* Trithorax [21], [22], [23], [24], [25], and regulates transcription by coordinating the methylation of histone H3K4 and the demethylation of H3K27 [26]. In addition, based on the recently established link between a super elongation complex and MLL, UTX might play a role as a general factor that is involved in the activation of gene transcription [25], [27]. Interestingly, sporadic mutations and the abnormal expression of UTX have been linked to many types of human cancers, suggesting that UTX plays a role in tumorigenesis. However, the functional role of UTX in tumorigenesis remains elusive. Because the DDR is generally accepted as a crucial safeguard against cancer, we hypothesize that UTX is involved in the DDR and plays an important role in maintaining genome integrity.

In this study, we demonstrated that UTX plays an essential role in the DDR in *Drosophila*. UTX is specifically required for the p53-dependent expression of *ku80* through mediating the demethylation of H3K27me3 upon exposure to ionizing radiation (IR). However, UTX is not required for the expression of other DNA repair genes, such as *ku70* and *mre11*, or the apoptotic genes *hid* and *reaper* (*rpr*). UTX is physically associated with p53, and IR exposure induces the recruitment of both UTX and p53 to the *ku80* promoter in an interdependent manner. These data favor a model in which UTX is a specific co-player in a p53-dependent cell survival response to DNA damage. Both UTX and p53 are functionally conserved from flies to humans. Therefore, our data demonstrate the role of UTX in the maintenance of genomic stability and might shed light on how UTX influences tumorigenesis.

## Materials and Methods

### *Drosophila* Genetics

All *Drosophila* lines were cultured in standard medium at 25°C. The P-element insertion mutant of *utx*, with a genotype of y1 w67c23; P{GSV6}GS10564/SM1, was obtained from the Drosophila Genetic Resource Center at the Kyoto Institute of Technology (http://kyotofly.kit.jp/cgi-bin/stocks). The P-element was mobilized using *P [delta 2–3]* as the source of P-element transposase according to standard protocols. A total of 176 independent white revertant lines were analyzed via PCR using genomic primers. One imprecise excision line, designated *utx^Δ95^* (containing a 1,691 bp deletion from ggttatttgtatgtatgtat to taaaccaatcagtgggcaat), was recovered. The *utx^1^* stock was kindly provided by Andreas Bergmann [28].

### Kc Cell Culture, RNAi knockdown and Transfection

Kc167 (Kc) cells were ordered from DRSC (Drosophila RNAi Screening Center) and were routinely cultured in Schneider’s Drosophila medium (Life Technologies) containing 5% FBS (Life Technologies) at 25°C. RNAi-mediated gene knockdown experiments were performed essentially as described previously [29]. Double-stranded RNAs (dsRNAs) targeting *control*, *utx* and *p53* sequences were synthesized as described elsewhere [26]. The following primer pairs were designed and used for the synthesis of the dsRNAs: *utx* forward, 5′-gaattaatacgactcactatagggagagagcaacaaaagttcggagc-3′; *utx* reverse, 5′-gaattaatacgactcactatagggagaatgaacagagggtgtgggag-3′; *p53* forward, 5′- gaattaatacgactcactatagggagaatcgtgggacagcatgttat-3′; and *p53* reverse, 5′- gaattaatacgactcactatagggagaaggctcagcaatttgttggc-3′; *ku80* RNAi forward, 5′-gaattaatacgactcactatagggagacgtgctctcgtttcttcgga-3′ and *ku80* RNAi reverse, 5′-gaattaatacgactcactatagggagaccgctctctttgacttctcc-3′. Five million cells were seeded in 25 cm^2^ flask with serum-free medium and incubated together with 37.5 µg of the dsRNAs (in equal amounts) for 30 min, and FBS was then supplemented at a 5% concentration. Total RNA was isolated two days later with the RNeasy mini kit (QIAGEN), and 2 µg of RNA was reverse transcribed using Superscript III reverse transcriptase (Life Technologies). We generated UTX mutant plasmid using QuikChange Lightning Site-Directed Mutagenesis Kit (Agilent technologies). The following primer pair is designed and used for the synthesis of *utx* mutant plasmid: *utx-j* mutant forward, 5′- cctggcgcgcaagcgaacaacaacttctgctcaatcaaca -3′ and *utx-j* mutant reverse, 5′-gttcgcttgcgcgccaggcgtcctacttccgggcaccttc-3′. For rescue experiment, we first treated the cells with dsRNA, and 2 days later re-plate the cells for transfection. Ten million cells were plated in 25 cm^2^ flask with medium containing 5% FBS and incubate at 25°C for 24 hours. Then each million cells transfected with 2 µg plasmid and X-tremeGENE Transfection Reagent (Roche). Incubated for 24 hours then following experiment.

### Ionizing Radiation (IR) and Survival Assay

For the IR and qRT-PCR experiments using Kc cells, γ-ray irradiation was applied to the cells at 2 or 4 days after RNAi treatment, and the cells were then harvested for total RNA extraction using the RNeasy mini kit (QIAGEN). To assess cell survival, RNAi-treated Kc cells were irradiated with 4 or 8 Gy of IR, seeded at a density of 1 × 10^6^ cells/ml into 6-well plates and counted at 2 and 4 days after irradiation. For hatching rate quantification, embryos were collected at 0–4 hours after embryo laying and irradiated with 10 Gy of IR, and the hatched larvae were counted after 3 days. For *utx^Δ95^*/*utx^1^* embryos, *utx^Δ95^*/*CyoGFP* flies were crossed with *utx^1^*/*CyoGFP* flies, and the non-fluorescent embryos were collected. For the third instar larva experiments, the total irradiation dosage was 40 Gy. Total RNA was isolated at 2 hours after irradiation using the RNeasy mini kit (QIAGEN).

### Real-time Quantitative RT-PCR Analysis

Real-time quantitative RT-PCR (qRT-PCR) assays were performed using the Applied Biosystems 7300 Real-Time PCR System (Life Technologies) and FastStart Universal SYBR Green Master Mix (Roche Applied Science). The following primer pairs were designed and employed for qRT-PCR: *ku80* forward, 5′- aagtccgcaaaatgtgtggc-3′; reverse, 5′- atttcatcggtgtcgcaacc-3′; *ku70* forward, 5′- cccatggtcgatgactttgac-3′; reverse, 5′- gaaaattgaacgccaaacagg-3′; *mre11* forward, 5′- ccaaaacggaggctgtcaat-3′; reverse, 5′- cgatccactaactcctccacg-3′; *hid* forward, 5′- cccaccgaccaagtgctatac-3′; reverse, 5′- ggcggatactggaagatttgc-3′; *rpr* forward, 5′- ccagttgttaattccgaacg-3′; reverse, 5′- tcgcctgatcgggtatgtaga-3′; *pnr* forward, 5′- gcaaggaggagcatgatctca-3′; reverse, 5′- tggtgccgctcttcatatcc-3′. *β-tubulin* levels were used as an internal control as described [29].

### Immunoprecipitation and Chromatin Immunoprecipitation (ChIP)

The immunoprecipitation experiments were performed with the PIERCE direct IP kit according to the manufacturer’s protocol using approximately 1×10^7^ cells. The ChIP assays were conducted using the Upstate ChIP assay kit, also following the manufacturer’s protocol. Briefly, approximately 3×10^7^ cells were collected, fixed and sonicated with a Bioruptor sonicator (Diagenode) to generate DNA fragments of approximately 500 bp in length. Next, immunoprecipitation was performed with either an antibody (3 µg) or normal rabbit IgG (3 µg), and the subsequent steps were performed as previously described [26]. The following primer pairs were used for qPCR: *ku80* forward, 5′-gcaacgcggtgctagaaatat-3′; reverse, 5′-gcggcttactgacctaatgca-3′; *ku70* forward, 5′-agcctgccgctgtaaaagtc-3′; reverse, 5′-accacctttcgatgacagcc-3′; *mre11* forward, 5′-cggtctatgtgatggcgaaat-3′; reverse, 5′-tgtcgtggtgccattcatg-3′; *hid* forward, 5′-agcaaaacaaagcagcgaaga-3′; reverse, 5′-tgctggcttcctttttgtcct-3′; *rpr* forward, 5′-cggcgtgagagaaccaggt-3′; reverse, 5′-ttttttcgagatgcgttcgc-3′; *pnr* forward, 5′- gcgttagccagcacaaagtg-3′; reverse, 5′-tggtgagcgaaagagcaaga-3′.

### Antibodies

Rabbit polyclonal anti-UTX antiserum was generated against a bacterially expressed GST-tagged UTX fragment (1–113aa). In the Western blot assays, the blots were first incubated with the proper concentrations of primary antibodies, followed by incubation with the indicated HRP-anti-rabbit IgG or HRP-anti-mouse IgG secondary antibodies (Sigma) and visualized using an ECL kit (Thermo Scientific). The antibodies used in the Western blot analysis were as follows: anti-UTX serum (1∶1,000), anti-β-Tubulin (1∶5,000, Sigma Cat. No. F1804) and anti-p53 (1∶1,000, DSHB Cat. No. 25F4); anti-UTX antiserum and anti-H3K27me3 (UPSTATE Cat. No. 07–449) were used for ChIP.

## Results

### The *utx* Gene is Essential for Cell Survival After IR Exposure

**Figure 1:**
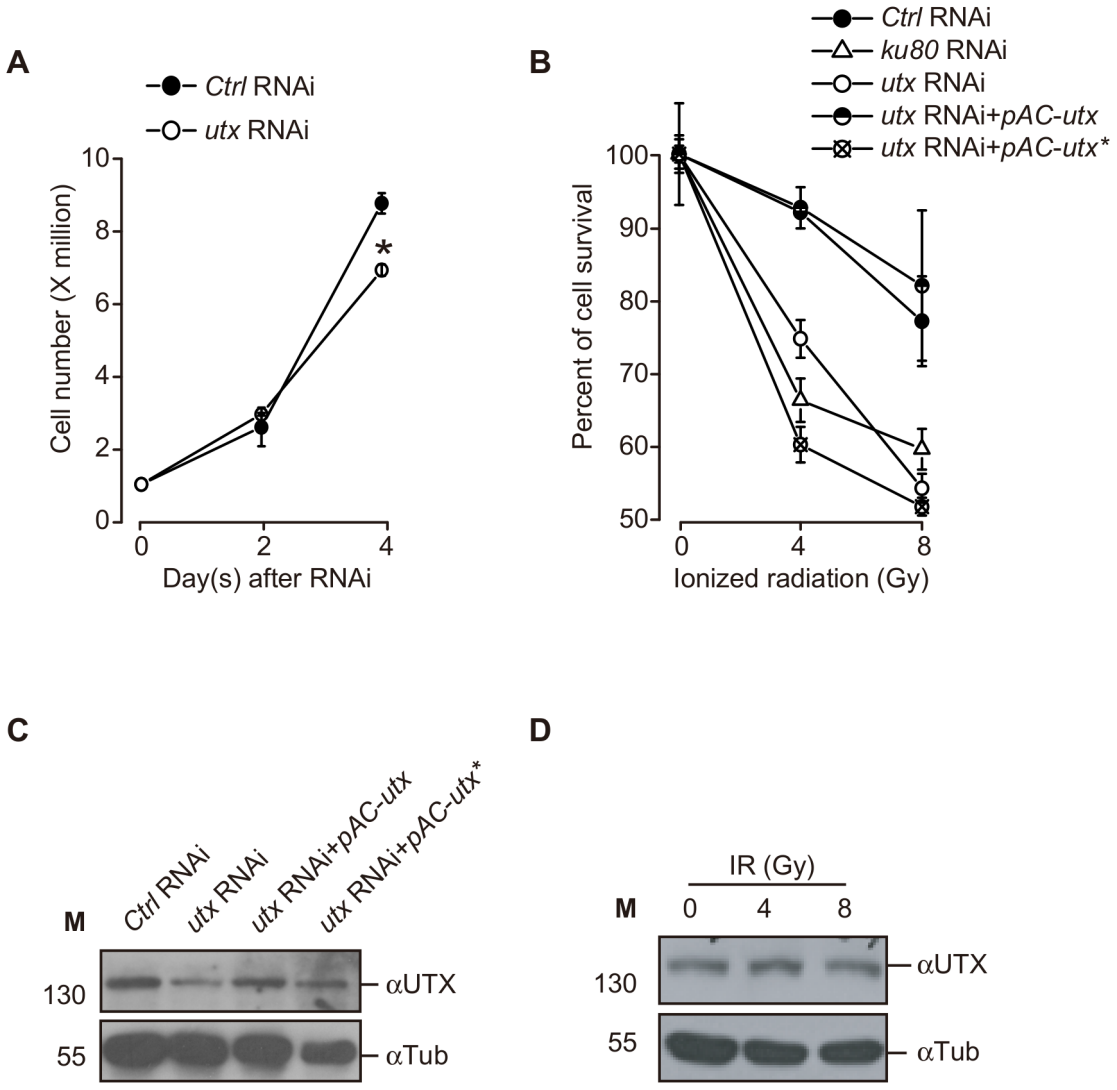
UTX is required for cell survival following IR exposure in Kc cells. (**A**) Cell counts at 2 and 4 days following RNAi treatment are indicated. Note that at 2 days, there is no difference in the growth rate of *utx* RNAi-treated compared to *control* (*Ctrl*) RNAi-treated cells. The asterisk indicates *P*<0.05 compared with the Ctrl RNAi group on the same day, as determined by two-way *ANOVA* followed by a *post-hoc* Tukey’s test for comparisons between groups. (**B**) *utx* RNAi-treated cells display significantly reduced cell counts compared to control RNAi-treated cells and over-expression wild type UTX could rescue RNAi effect but not mutant UTX after IR exposure. 4 days RNAi-treated cells were irradiated at doses of 4 and 8 Gy and examined relative cell viability after 2 days.(**C**) Western blot analysis of UTX expression confirming the efficiency of *utx* RNAi-mediated knockdown and over-expression of UTX. (**D**) IR treatment causes no detectable change in UTX protein levels. β-Tubulin (Tub) levels were used as loading controls. All of the data are representative of at least three independent experiments with similar results. Error bars indicate standard deviations from triplicate sets of the presented experiment.

The UTX protein has been extensively studied regarding its function as a demethylase and its H3K27 demethylase-independent activity during development [15], [18], [28]. Previous studies indicate that UTX is also associated with human cancers [30], [31]. However, how UTX functions as a tumor suppressor is unclear. For this reason, we hypothesized that UTX is involved in the maintenance of genomic stability in response to DNA damage, as loss of UTX function results in genome instability and tumorigenesis. Under basal conditions, the RNAi-mediated depletion of *utx* did not affect the growth of Kc cells at 2 days after soaked with double-strend RNAs and caused only a slight slower growth at 4 days (Fig. 1A). We challenged 4 days RNAi-treated cells with IR at doses of 4 and 8 Gy and examined relative cell viability after 2 days. We found that both doses of IR induced a significant reduction in the viability of the cells treated with *utx* RNAi compared to controls RNAi cells. The reduction of cell survival is indeed due to the loss of *utx*, since over-expression of wild type UTX in RNAi cell could rescue the viability (Fig. 1B). UTX protein levels were significantly reduced after the RNAi-mediated knockdown of *utx* but remained steady after IR, suggesting that IR does not regulate the expression of UTX (Fig. 1C, 1D). These data indicate that UTX is required for cell survival/growth upon genotoxic insult in Kc cells.

### UTX Upregulates *ku80* Through Promoter Demethylation in Response to IR Exposure

**Figure 2:**
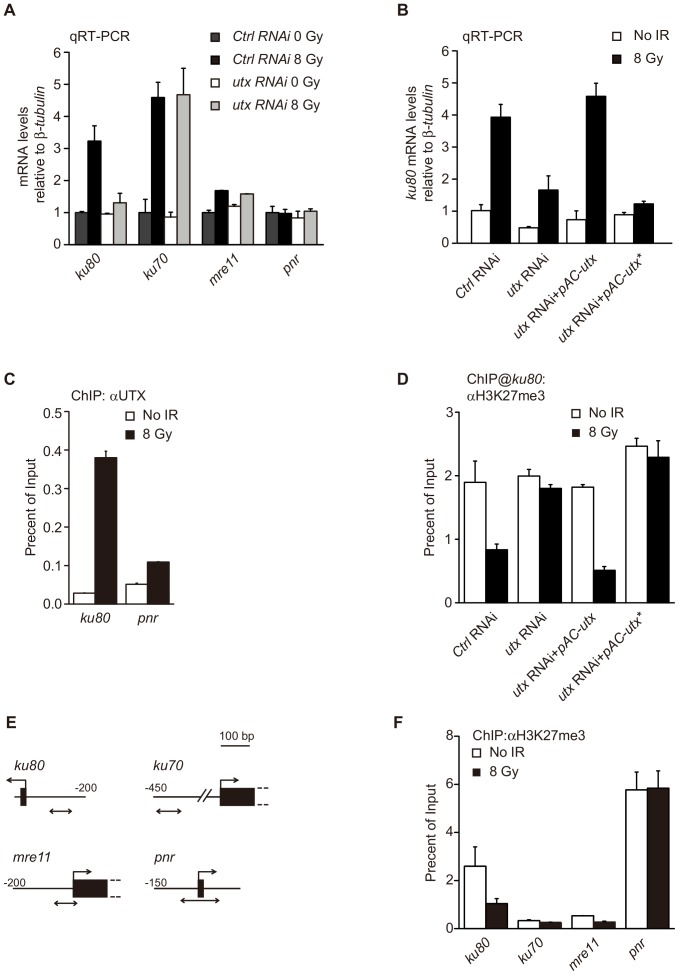
UTX is required for the expression of *ku80* after IR exposure *via* the demethylation of H3K27me3 in Kc cells. (**A**) qRT-PCR analysis of the mRNA expression of the indicated genes before and after IR exposure in RNAi-treated Kc cells. The relative expression levels are normalized to β-*tubulin* levels. Note that *ku80* is the only gene that requires *utx* for its expression. (**B**) qRT-PCT analysis of *ku80* expression in different treatment cell. Over-expression WT UTX could rescue *ku80* expression in UTX RNAi cell after IR but not mutant UTX. (**C**) ChIP assay with an anti-UTX antibody and the *ku80* promoter with and without IR. Note the dramatic increase in the UTX occupancy of the *ku80* promoter after IR. (**D**) ChIP assay for H3K27me3 at the *ku80* promoter in different treatment Kc cells after IR. (**E**) The diagrams show PCR-amplified regions (double arrows) relative to the first exons (black box) in the ChIP analysis of the three DNA repair genes *ku80*, *ku70* and *mre11* and a control gene, *panier* (*pnr*). (**F**) Changes in H3K27me3 levels at the indicated genes 2 hours after IR treatment with a dose of 8 Gy. The H3K27me3 levels in the promoter regions of those genes were determined *via* ChIP assays and compared to the input genomic DNA.

In a previous microarray analysis that conducted in our laboratory, we identified a list of genes that are upregulated following IR treatment in *Drosophila* Kc167 (Kc) cells (Table S1). This list included genes known for their roles in DSB repair, such as *ku70*, *ku80* and *mre11*, similar to what has been previously reported in fly embryos [32]. To further determine the role of UTX in the DDR, we investigated whether UTX is involved in the regulation of these genes in response to IR exposure. Interestingly, we found that the RNAi-mediated knockdown of UTX expression significantly inhibited the upregulation of *ku80*, but not that of *ku70* and *mre11*, following IR treatment (Fig. 2A). Over-expression of wild type UTX in *utx* RNAi cell restored the upregulation of *ku80* expression (Fig. 2B). These data suggest that UTX regulates *ku80* expression in a gene-specific manner in DDR. This notion is further supported by the fact that both *utx and ku80* RNAitreated cells showed similar cell sensitivity to IR (Fig. 1B). Next, we explored whether UTX regulates directly or indirectly the expression of *ku80* upon IR treatment. Chromatin immunoprecipitation (ChIP) assays revealed that UTX is recruited to the promoter region of *ku80* upon IR treatment (Fig. 2C). Although UTX has predominantly been shown to regulate transcription by demethylating H3K27, it has also been found that UTX regulates the mesoderm differentiation of embryonic stem cells, independent of its H3K27 demethylase activity in mouse [33]. Therefore, we investigated whether the observed UTX-mediated *ku80* expression was dependent on the demethylase activity of UTX.

Using ChIP assays, we found that IR treatment dramatically reduced the levels of H3K27me3 in the *ku80*, but not in *utx* depleted cells. Over-expression WT UTX could reduce the levels of H3K27me3 in *utx* RNAi cells after IR, but not JMJC domain mutant UTX which disrupt UTX enzyme activity (Fig. 2D) [28]. Furthermore, we found that over-expression JMJC domain mutant UTX could not rescue cell survival and *ku80* expression compare with WT UTX in *utx* RNAi cell after IR (Fig. 1B, Fig. 2B). These data indicate that UTX functions as a histone demethylase in its regulation of *ku80* upon IR exposure. To further assess whether DNA damage genes are activated in association with the altered levels of H3K27me3, we examined the promoter regions of these genes before and after IR treatment. We found that IR treatment induced a dramatic reduction of H3K27me3 levels in *ku80*, but not in *ku70* or *mre11* (Fig. 2E, 2F). As a control gene, we also examined a known Polycomb target gene, *pannier* (*pnr*), and we found that IR caused no apparent changes in either the expression or the recruitment of UTX to *pnr* [26]. These data indicate that during DNA damage, UTX specifically and directly regulates the expression of *ku80* by demethylating histone H3K27me3 at the *ku80* promoter.

### UTX Coordinates with p53 to Directly Facilitate the Expression of *ku80* Following IR Treatment

**Figure 3:**
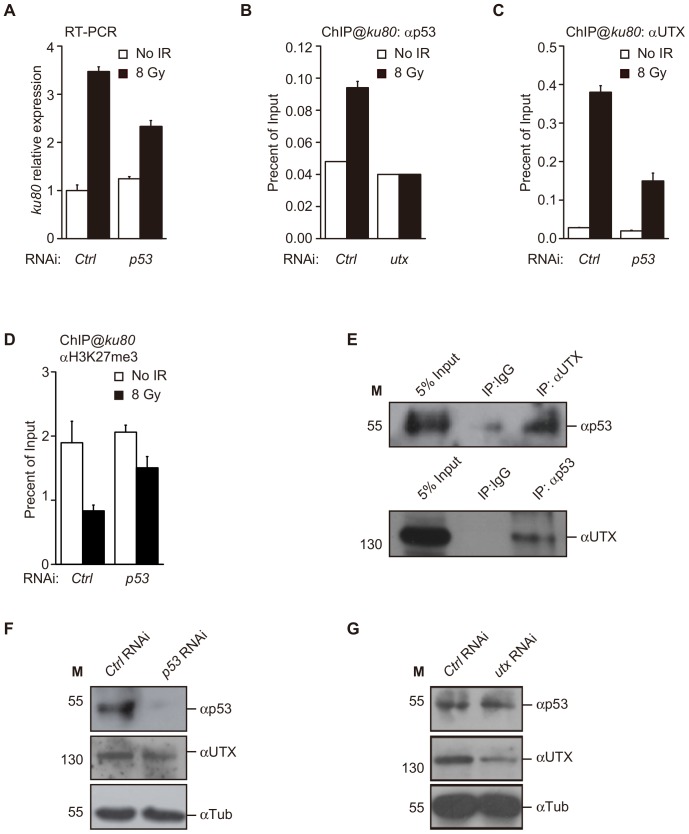
p53 and UTX are recruited in an interdependent manner to the *ku80* promoter region. (**A**) *ku80* expression following IR exposure in Kc cells subjected to RNAi treatment, as indicated. (**B, C**) ChIP analysis of the physical occupancy of p53 and UTX at the *ku80* promoter region. Note that knockdown of *utx* eliminates the increase in p53 binding, and knockdown of *p53* reduces the binding of UTX to the *ku80* promoter. (**D**) ChIP assay for H3K27me3 at the *ku80* promoter in Kc cells treated with control or *utx* RNAi after IR. (**E**) Coimmunoprecipitation was performed using anti-p53 and anti-UTX antibodies and whole cell extracts of Kc cells. The immunoprecipitates were subjected to Western blot analysis with the indicated antibodies. (**F, G**) Western blot analysis to confirm the knockdown efficiency of *p53* RNAi. β-Tubulin (β-Tub) levels were used as a loading control.

Previous studies have indicated that *ku80*, as well as *ku70*,is among the p53 target gene list in fly embryos [32] and larvae [34]. In Kc cells, we found that the expression of *ku80* also requires p53, as the RNAi-mediated knockdown of *p53* significantly reduced the expression of *ku80* following IR treatment (Fig. 3A). In addition, using ChIP analysis, we showed that IR caused marked p53 enrichment in the *ku80* promoter (Fig. 3B). Next, we asked whether UTX and p53 coordinate their activities to regulate the expression of *ku80*. The RNAi-mediated knockdown of UTX expression significantly inhibited the recruitment of p53 to the *ku80* promoter, suggesting that UTX is required for the regulation of *k80* expression by p53 (Fig. 3C). Intriguingly, we found both the UTX recruitment and the reduction of H3K27me3 levels in the *ku80* gene were also prevented by the knock-down of *p53* (Fig. 3C, 3D). These data indicate that both p53 and UTX directly regulate *ku80* expression within the same pathway, thus requiring coordinated action between p53 and UTX. Further supporting this notion, we found that p53 coimmunoprecipitated with UTX, and UTX was similarly able to coimmuniprecipitate with p53, indicating a physical interaction between the two proteins (Fig. 3E). However, we did not observe a direct interaction in GST pull-down assays, suggesting that the interaction between p53 and UTX is indirect. The UTX protein level is not affected by the change of p53 level as confirmed by the Western blot (Fig. 3F). Similarly, UTX does not regulate p53 expression (Fig. 3G). These data exclude the possibility that UTX and p53 interacts in DDR by the mutual regulation of expression. Together, these data support a molecular model in which p53 and UTX form a complex to regulate *ku80* expression and mediate the DDR following exposure to IR.

### UTX Regulates *ku80* Expression in *Drosophila*

**Figure 4:**
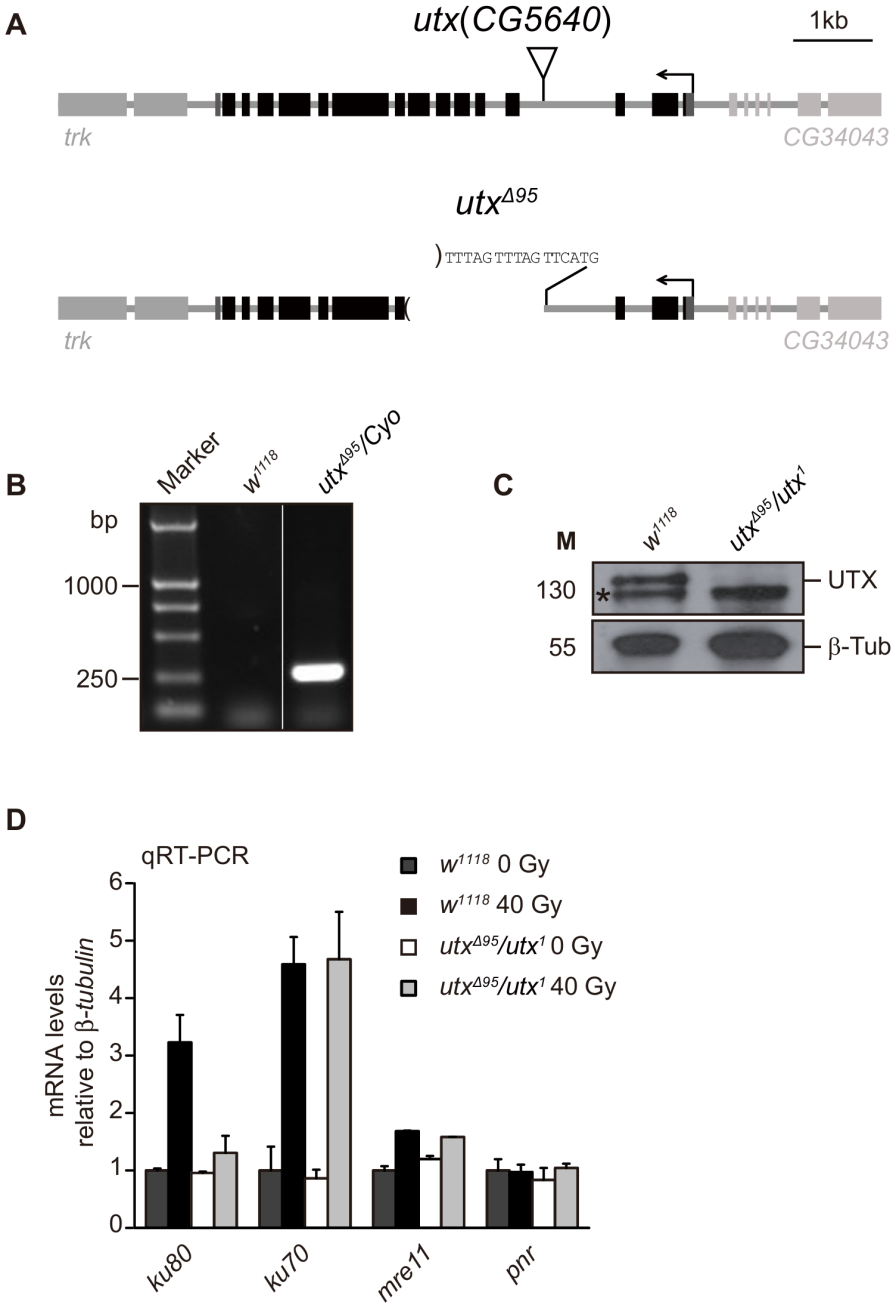
UTX is required for the expression of *ku80* following IR exposure in *Drosophila.* (**A**) Schematic illustration of the gene structure of wild type *utx* and a *utx* mutant allele (*utx^Δ95^*) generated *via* the imprecise excision of a P-element insertion. (**B, C**) Genomic PCR (B) and Western blot (C) analyses to verify the *utx^Δ95^* genotype. (B) An approximately 250-bp band is detected in adult flies with a *utx^Δ95^*/C*yo* genotype, but absent from the *w^1118^* genotype. For details, please see the Materials and Methods section. (C) An approximately 130-kDa band indicated by an arrow in *w^1118^* flies was not detected in the *utx^Δ95^*/*utx^1^* third instar larvae. A non-specific band is indicated by an asterisk, and β-Tubulin (β-Tub) was used as a loading control. (**D**) qRT-PCR analysis of mRNA expression for the indicated genes before and after IR exposure in the *w* ^1118^ and *utx^Δ95^*/*utx^1^* third instar larvae. The relative expression levels are normalized to β-*tubulin* levels. Note that *ku80* is the only gene that requires *utx* for its expression.

Using *Drosophila* as an *in vivo* model system, we next investigated whether the expression of *ku80* is also regulated by UTX in *Drosophila*. To address this question, we generated a *utx* mutant allele, *utx^Δ95^*, through the imprecise excision of a P-element inserted into the *utx* locus (Fig. 4A). Genomic PCR analysis and sequencing data confirmed the existence of a deletion of five exons (1,691 base pairs) in *utx^Δ95^*, as indicated by FlyBase gene annotation (http://flybase.org/) (Figs. 4A & 4B). We found that animals homozygous for *utx^Δ95^* only rarely survive to adults but *utx^Δ95^/utx^1^* trans-heterozygotes can develop into adults and show no detectable morphological defects. Those results consistent with recently published article [35]. A reported EMS allele, *utx^1^*, bearing a nonsense mutation in the JmjC domain, has also been used [28]. Both *utx* alleles are null, as verified by the missing UTX band in a Western blot analysis of trans-heterozygous (*utx^Δ95^/utx^1^*) third instar larvae performed using an anti-UTX antiserum raised against the N-terminal 103 aa portion of the protein (Fig. 4C). As shown in Figure 4D, 8 Gy of IR dramatically upregulated the levels *ku80*, *ku70* and *mre11* in *w^1118^* (wild type control) third instar larvae, similar to what was observed in Kc cells (Fig. 2A), indicating the expression of these genes upon DNA damage in *Drosophila*. However, IR treatment elicited a significantly reduced induction of *ku80* expression in *utx^Δ95^/utx^1^* third instar larvae compared to wild type larvae, whereas the expression of other genes remained relatively constant, suggesting an gene-specific requirement of UTX for *ku80* expression during the DDR. We therefore conclude that UTX is essential for the expression of *ku80* both in cell and *Drosophila*.

Furthermore, to determine whether UTX is involved in DNA repair in *Drosophila*, we quantified the hatching rate of transheterozygous *utx* null (*utx^Δ95^*/*utx^1^*) and *w^1118^* embryos treated with IR. The *utx* null embryos exhibited a markedly lower hatching rate of 69.6% compared to the wild type embryos, which displayed a hatching rate of 96.0% (Table 1). Treatment with 10 Gy of IR severely reduced the hatching rate for both genotypes. However, we conclude that the effect of IR was more significant for *utx* null embryos, as demonstrated by the statistically significant reduction of the normalized hatching rate (Table 1). These data suggest that *utx* mutant embryos are more sensitive to IR stress than wild type embryos. In addition, we found that the hatching rate of *Drosophila* embryo does not significantly change between wild type and mutant after UV irradiation (Table 2). Therefore, UTX might play an essential role in DNA repair through regulating *ku80* expression both in cell and *Drosophila*.

**Table 1:** The hatching rate of *Drosophila* embryo.

	γ-ray irradiation			Normalized hatching rate	
Genotype	0 Gy	5 Gy	10 Gy	5 Gy	10 Gy
*w^1118^*	499/520 (96.0%)	98/185 (53.0%)	28/166 (16.9%)	55.3%	17.6%
*utx^Δ95^/utx^1^*	188/270 (69.6%)	42/127 (33.1%)	7/170 (4.0%)	47.7% a	5.7% ^b^

a *P*>0.05 and ^b^ *P*<0.01, as analyzed by *Fisher’s Exact Test* for normalized hatch rate upon IR treatment of 0–4 hrs *w ^1118^* and *utx^Δ95^/utx^1^* embryo.

**Table 2:** The hatching rate of *Drosophila* embryo.

	UV irradiation			Normalized hatching rate	
Genotype	0 J/m^2^	10 J/m^2^	100 J/m^2^	10 J/m^2^	100 J/m^2^
*w^1118^*	499/520 (96.0%)	124/220 (56.4%)	93/205 (45.4%)	58.8%	47.3%
*utx^Δ95^/utx^1^*	188/270 (69.6%)	55/145 (37.9%)	69/179 (38.5%)	54.5% a	55.3% a

a *P*>0.05, as analyzed by *Fisher’s Exact Test* for normalized hatch rate after 48 hrs upon UV treatment of 0–4 hrs *w ^1118^* and *utx^Δ95^/utx^1^* embryo.

### UTX is Not Responsible for the Upregulation of Apoptotic Genes in Response to DNA Damage

**Figure 5:**
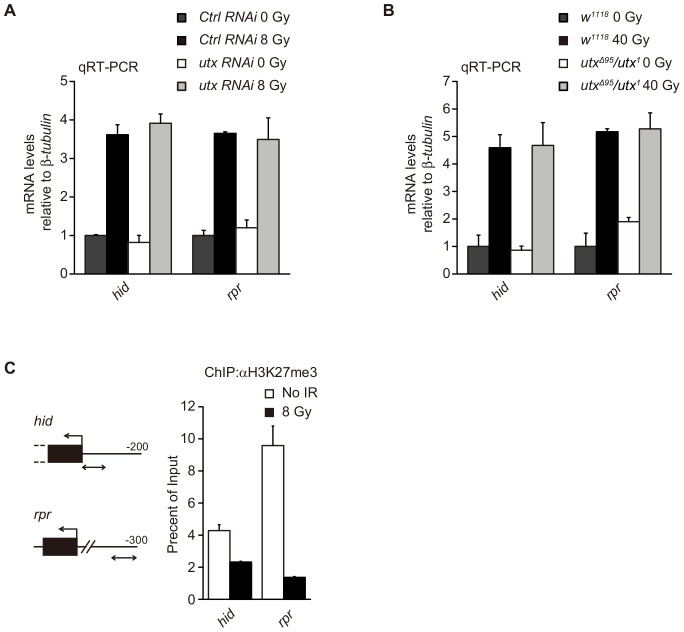
UTX is not responsible for the upregulation of apoptosis-related genes following IR exposure. (**A, B**) qRT-PCR analysis of the mRNA expression of the indicated genes before and after IR treatment in RNAi-treated Kc cells, as shown for *w^1118^* and *utx^Δ95^*/*utx^1^* third instar larvae. The relative expression levels are normalized to β-*tubulin* levels. (**C**) The diagrams show PCR-amplified regions (double arrows) relative to the first exons (black box) in the ChIP analysis for two apoptosis-related genes, *hid* and *rpr*. The changes in H3K27me3 levels at the genes are indicated 2 hours after IR treatment with a dose of 8 Gy.

Previous studies have shown that p53 upregulates the apoptotic genes *reaper* and *hid* following treatment with IR [32]. Given that together with p53, UTX coordinately regulates the expression of *ku80* upon IR exposure, we sought to determine whether UTX also participates in the regulation of apoptotic gene expression in response to DNA damage. To investigate this notion, we evaluated the changes in *reaper* and *hid* expression levels following IR treatment. Figures 5A and 5B show that independent of UTX, the expression levels of these apoptotic genes were upregulated to the same extent following IR exposure both in cell and *Drosophila*. These results were consistent with the findings of previous works showing that overexpression of UTX in primary human fibroblasts induces cell cycle arrest, but not apoptosis [36]. These data suggest that p53, but not UTX, is required for DNA damage-induced apoptosis. Interestingly, we found that the levels of H3K27me3 at the *reaper* and *hid* promoters were also reduced following IR treatment, similar to what was observed for the *ku80* promoter (Fig. 5C). These data suggest that other demethylases might be responsible for the upregulation of apoptotic genes in response to DNA damage.

## Discussion

To understand the mechanism underlying UTX function in tumorgenesis, we explored whether UTX is involved in DNA damage response in *Drosophila*. In this study, we found that UTX, play an essential role in DNA damage response by upregulation of *ku80*, which is uniquely required for p53 activated *ku80* expression (Fig. 2–5). In addition, the gene activity of *utx* is correlated with loss of histone demethylation at H3K27 (Fig.2), suggesting that UTX could function as a histone demethylase and serve a gene-specific co-activator of p53 gene activation. We therefore provide an example that p53 target genes expression may be regulated at the level of histone modifications.

**Figure 6:**
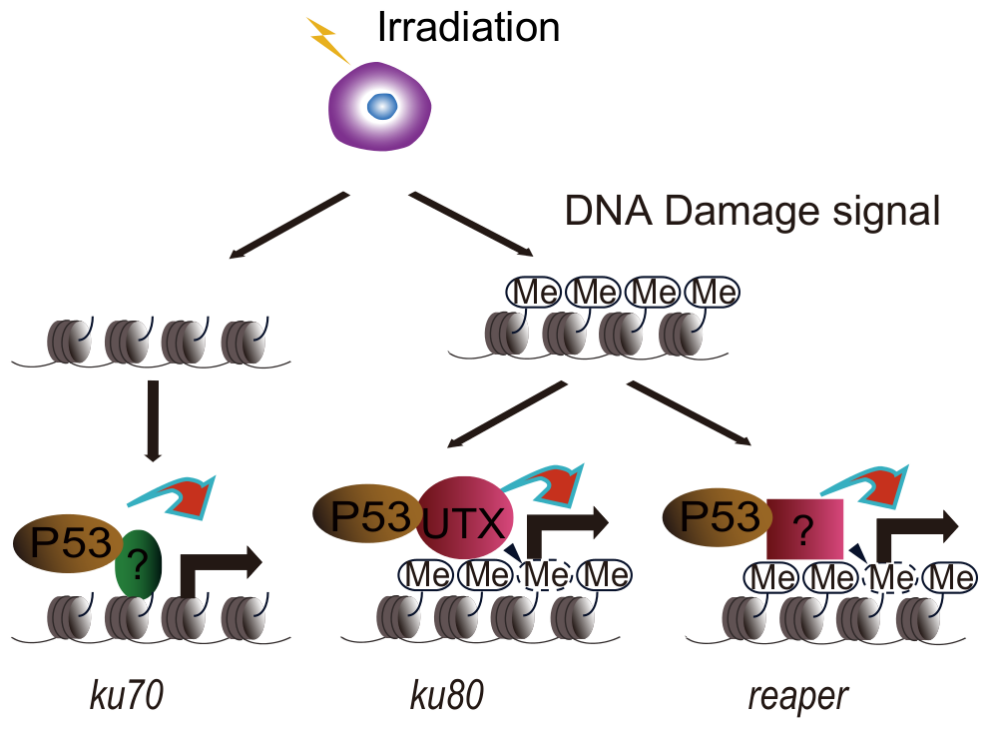
A model for the regulation of DNA damage response genes associated with DNA double-strand breaks in cells is suggested based on the results presented in **Figures 2**–**5**. See text.

It is clear that p53 plays a pivotal role in the DNA damage response (DDR). One of the functions of p53 is to activate its target gene after DNA damage as transcription factor. For instance, p53 has been best characterized in regualting expression of cell cycle genes and apoptosis gene [37]. However, the precise reguation mechnism of p53 is still not clear. It is interesting that in *Drosophila ku80* upregulation mediated by p53 requires UTX, but not other genes in related to DNA repair and apoptosis. However, we did observe reduced H3K27me3 levels in apoptotic genes (Fig.2), which raise the possibility that there could be additional histone demethylases participating in DDR pathways that coordinate with p53 regulating expression of *hid* and *reaper* after DNA damage, and remaining to be determined in further studies. In contrast, we did not detect reduced H3K27me3 levels in the *ku70* promoter region following IR treatment. Further analysis revealed that the H3K27me3 level in the *ku70* promoter region was lower than at the *ku80* promoter. The expression of *ku70* is independent of UTX, possibly due to the extremely low levels of H3K27me3 in the *ku70* promoter region, which might not require demethylation for the expression of *ku70* to occur (Fig. 2F). Thus, our data demonstrate the complexity of the function of p53 in the activation of target genes in response to DNA damage, particularly in terms of histone modification and the action of different demethylases (Fig. 6).

UTX has been reported to participate in many biological processes, including cell fate determination and animal development [15], [18], [28], [36], [38], largely depending on the transcriptional regulation of the target genes of UTX. UTX appears to play an important role in orchestrating several histone marker, including acetylation at H3K27 and ubiquitination at H2A [19], [39], [40], and mediates derepression of polycomb (Pc) target genes, such as HOX genes, by affecting Pc recruitment. These roles are consistent with UTX being a histone demethylase specific for H3K27 [20]. However, sporadic mutations of UTX have been linked to many types of human cancers [30], [41], [42] and it remains to be elucidated whether this is also sufficiently explained by its enzymatic activity. Indeed, several studies have proposed a role of UTX independent of its demethylase activity in chromatin remodeling and embryonic development [33], [43], [44]. In this study, we found UTX is also involved in DDR by upregulation of *ku80* in *Drosophila* after IR. Although there are no available data demonstrating that *ku80* mRNA levels are increased following DSBs in human cells, our data provide evidence that UTX functions to maintain genome stability and shed light on the mechanism underlying the function of UTX in human cancer. Recent studies suggest that loss of polycomb-mediated silencing might promote the upregulation of DNA repair genes [45] and facilitate the recovery of cells from genotoxic insults. UTX might therefore be required for various cell defense mechanisms under environmental stress, thereby contributing to tumor suppression.

## Supporting Information

Figure S1 qRT-PCR analysis to confirm the knockdown efficiency of ku80 RNAi.
(TIF)

Table S1 Contains partial of microarray data which shows fold change more than five times of genes expression up-regulated following IR.
(DOCX)

Text S1 Only contains the legend for Figure S1 and is not intended for publication.
(DOC)
