# Supplementary figures and images for: Drosophila UTX Coordinates with p53 to Regulate ku80 Expression in Response to DNA Damage
Source: PLoS One. 2013 Nov 12;8(11):e78652. doi: 10.1371/journal.pone.0078652 (PMC3827076; doi:10.1371/journal.pone.0078652)

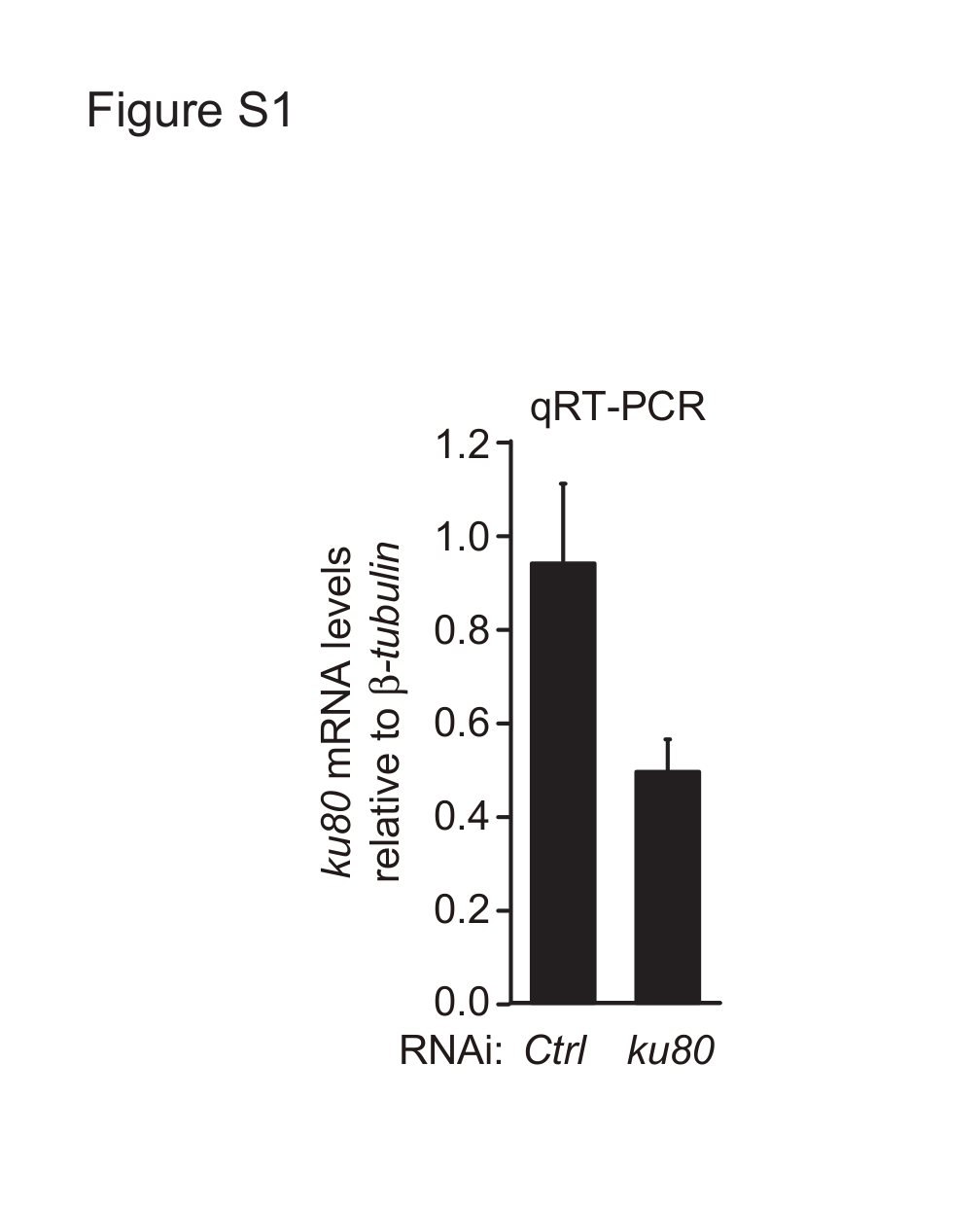

Supplement: Figure S1 — qRT-PCR analysis to confirm the knockdown efficiency of ku80 RNAi. (TIF) [file pone.0078652.s001.tif]
