# Supplementary material for: Drosophila UTX Coordinates with p53 to Regulate ku80 Expression in Response to DNA Damage
Source: PLoS One. 2013 Nov 12;8(11):e78652. doi: 10.1371/journal.pone.0078652 (PMC3827076; doi:10.1371/journal.pone.0078652)
Supplement: Table S1 — Contains partial of microarray data which shows fold change more than five times of genes expression up-regulated following IR. (DOCX) [file pone.0078652.s002.docx]

Table S1. Gene expression up-regulated following IR

| Gene | Fold change | Gene | Fold change | Gene | Fold change |
| --- | --- | --- | --- | --- | --- |
| CG6044 | 25 | CG6508 | 8.5 | CG33475 | 6.2 |
| Os-C | 22.6 | CG17377 | 8.5 | CG12029 | 6.2 |
| Cyp318a1 | 22.4 | CG34045 | 8.5 | CG31730 | 6.2 |
| CG4375 | 21.1 | CG6784 | 8.5 | CG12995 | 6.2 |
| htl | 20.8 | CG2983 | 8.5 | CG7365 | 6.2 |
| CG8394 | 20.8 | CG4483 | 8.5 | CG32026 | 6.2 |
| CG31051 | 20.2 | Sox102F | 8.5 | PH4alphaSG2 | 6.2 |
| CG15685 | 18.9 | CG7708 | 8.5 | CG6888 | 6.2 |
| CG16735 | 18.7 | CG30430 | 8.5 | CG5391 | 6.2 |
| MtnB | 18.3 | byn | 8.5 | CG7320 | 6.2 |
| CG32106 | 18.2 | CG16723 | 8.5 | CG11458 | 6.1 |
| obst-H | 17.5 | CG5022 | 8.4 | CG10104 | 6.1 |
| Dr | 17.4 | bol | 8.4 | CG7763 | 6.1 |
| Dgk | 16.7 | CG32626 | 8.4 | CG34336 | 6.1 |
| Tektin-A | 16.3 | BobA | 8.4 | Hr46 | 6.1 |
| CG34212 | 15.8 | CG13465 | 8.4 | CG31533 | 6.1 |
| CG17234 | 15.8 | CG14142 | 8.4 | TwdlF | 6.1 |
| CG31851 | 15.8 | CG32298 | 8.4 | CG15800 | 6.1 |
| trp | 15.3 | CG32299 | 8.4 | CG13108 | 6.1 |
| CG14870 | 15.3 | CG15406 | 8.4 | CG13795 | 6.1 |
| Rapgap1 | 15 | CG34047 | 8.4 | salm | 6.1 |
| CG32334 | 14.8 | CG32006 | 8.4 | CG6036 | 6.1 |
| CG13506 | 14.6 | CG32396 | 8.3 | CG13337 | 6.1 |
| hb | 14.5 | CG42235 | 8.3 | CG34024 | 6.1 |
| grh | 14.5 | tau | 8.3 | CG13331 | 6.1 |
| CG14579 | 14.5 | CG15449 | 8.3 | CG17855 | 6.1 |
| CG11664 | 14.4 | bap | 8.2 | CG13049 | 6.1 |
| CG9259 | 14.3 | Cpr76Bc | 8.2 | CG33281 | 6.1 |
| CG34209 | 14.1 | CG9080 | 8.2 | CG31630 | 6 |
| CG33232 | 14.1 | CG33346 | 8.2 | lectin-37Db | 6 |
| CG4829 | 14 | cad | 8.2 | CG17196 | 6 |
| Cpr97Eb | 14 | oa2 | 8.2 | CG5568 | 6 |
| CG11147 | 13.8 | Nmdar1 | 8.2 | CG18586 | 6 |
| dpr2 | 13.7 | CG33200 | 8.1 | CG4623 | 6 |
| CG30101 | 13.7 | CG3790 | 8.1 | dpn | 6 |
| CG13039 | 13.6 | CG3349 | 8.1 | cpo | 6 |
| CG4734 | 13.6 | CG18539 | 8.1 | CG8916 | 6 |
| Odc2 | 13.5 | CG8252 | 8.1 | CG17819 | 6 |
| CG14537 | 13.2 | kay | 8 | CG15785 | 6 |
| CG9400 | 13.2 | CG33988 | 8 | CG15786 | 6 |
| CG15460 | 13.1 | Fas2 | 8 | Andorra | 5.9 |
| Obp19a | 13.1 | alpha-catenin-related | 8 | CG17244 | 5.9 |
| CG32987 | 13.1 | ey | 8 | Obp8a | 5.9 |
| CG13801 | 12.9 | CG11910 | 8 | fzy | 5.9 |
| CG11878 | 12.8 | CG3483 | 7.9 | HLH4C | 5.9 |
| CG40298 | 12.7 | CG13921 | 7.9 | CG7896 | 5.9 |
| RpL10Aa | 12.7 | dpr10 | 7.9 | C15 | 5.9 |
| CG13972 | 12.7 | CG32033 | 7.9 | CG17944 | 5.8 |
| CG14492 | 12.7 | CG15625 | 7.8 | CG12003 | 5.8 |
| CG15035 | 12.5 | CG33958 | 7.8 | CG30071 | 5.8 |
| CG33934 | 12.5 | CG9589 | 7.8 | CG9497 | 5.8 |
| CG42234 | 12.4 | Or65c | 7.8 | CG32463 | 5.8 |
| CG17111 | 12.4 | CG14431 | 7.8 | CG8236 | 5.8 |
| CG42255 | 12.2 | ninaD | 7.8 | CG30194 | 5.8 |
| Lcp65Ag3 | 12.1 | CG3822 | 7.7 | Mf | 5.8 |
| ftz | 12.1 | CG30181 | 7.6 | CG9235 | 5.8 |
| CG32192 | 12.1 | CG3746 | 7.6 | CG13932 | 5.8 |
| CG14455 | 12 | CG34351 | 7.6 | CG17198 | 5.8 |
| CG34356 | 12 | pwn | 7.6 | CG3769 | 5.7 |
| dpr3 | 12 | CG12586 | 7.6 | CG9170 | 5.7 |
| pb | 11.9 | CG6361 | 7.6 | X11Lbeta | 5.7 |
| CG40116 | 11.9 | CG13616 | 7.6 | CG2104 | 5.7 |
| CG13306 | 11.9 | CG8086 | 7.6 | Gr89a | 5.7 |
| CG12187 | 11.8 | CG17364 | 7.6 | CG12209 | 5.7 |
| CG1698 | 11.8 | exex | 7.6 | Nmdar2 | 5.7 |
| CG7017 | 11.8 | CG18869 | 7.5 | CG31526 | 5.7 |
| CG42368 | 11.8 | CG15594 | 7.5 | CG31423 | 5.7 |
| CG34274 | 11.7 | CG3649 | 7.5 | CG30376 | 5.7 |
| obst-J | 11.7 | CG14853 | 7.5 | CG11206 | 5.6 |
| CG12849 | 11.7 | CG10749 | 7.5 | CG11293 | 5.6 |
| Porin2 | 11.6 | CG15233 | 7.5 | CG14105 | 5.6 |
| krz | 11.6 | CG30461 | 7.5 | Taf12L | 5.6 |
| CG42377 | 11.6 | CG9002 | 7.5 | CG34181 | 5.6 |
| syt | 11.6 | w-cup | 7.4 | zen | 5.6 |
| CG1887 | 11.5 | CG32186 | 7.4 | CG17906 | 5.6 |
| CG12436 | 11.4 | CG7349 | 7.4 | CG7164 | 5.5 |
| CG1090 | 11.4 | Cyp317a1 | 7.4 | Cpr11B | 5.5 |
| CG13293 | 11.3 | CG14186 | 7.4 | SIFR | 5.5 |
| Osi12 | 11.3 | CG34253 | 7.4 | CG34041 | 5.5 |
| CG8743 | 11.3 | CG16727 | 7.4 | CG13229 | 5.5 |
| yin | 11.3 | CG40486 | 7.4 | CG7509 | 5.5 |
| CG16884 | 11.3 | tsg | 7.3 | CG40203 | 5.5 |
| CG4395 | 11.2 | CG34384 | 7.3 | CG9313 | 5.5 |
| CG42342 | 11.1 | Lsp1alpha | 7.2 | CG18367 | 5.5 |
| CG10877 | 11.1 | CG30037 | 7.2 | Lcp65Ae | 5.5 |
| CG13405 | 11.1 | Ace | 7.2 | CG7882 | 5.5 |
| CG30463 | 11.1 | CG12424 | 7.2 | CG10361 | 5.5 |
| CG11113 | 11.1 | CG15865 | 7.2 | up | 5.5 |
| CG13713 | 11.1 | CG1850 | 7.2 | CG31530 | 5.4 |
| CG7856 | 11 | CG13924 | 7.1 | CG7367 | 5.4 |
| CG13133 | 11 | CG2267 | 7.1 | CG30384 | 5.4 |
| CG6865 | 11 | CG30160 | 7.1 | CG30385 | 5.4 |
| CG6136 | 11 | Tsp42Eb | 7.1 | CG15036 | 5.4 |
| CG11629 | 10.9 | CG34109 | 7.1 | CG7248 | 5.4 |
| CG33221 | 10.9 | Or2a | 7.1 | CG5204 | 5.4 |
| CG18558 | 10.9 | CG12535 | 7.1 | CG31826 | 5.4 |
| Or49a | 10.9 | CG8785 | 7.1 | Ku80 | 5.4 |
| trio | 10.9 | CG31031 | 7.1 | CG16716 | 5.4 |
| CG16848 | 10.9 | nec | 7.1 | CG41106 | 5.4 |
| TwdlZ | 10.8 | Or63a | 7 | skpF | 5.4 |
| CG32588 | 10.7 | CG3557 | 7 | CG30053 | 5.3 |
| CG42249 | 10.6 | Jheh3 | 7 | CG12116 | 5.3 |
| CG34028 | 10.5 | retinophilin | 6.9 | CG12831 | 5.3 |
| CG6332 | 10.4 | CG31556 | 6.9 | CG31097 | 5.3 |
| Cpr65Eb | 10.4 | CG32118 | 6.9 | CG12963 | 5.3 |
| Vha100-3 | 10.4 | CG10226 | 6.9 | Ilp3 | 5.3 |
| CG13438 | 10.4 | CG3259 | 6.9 | fs(1)N | 5.3 |
| CG11630 | 10.3 | phm | 6.9 | Fili | 5.3 |
| CG31025 | 10.3 | CG32193 | 6.9 | CG6465 | 5.3 |
| Or69a | 10.3 | B-H1 | 6.9 | CG5357 | 5.3 |
| wa-cup | 10.2 | CG18234 | 6.8 | Gr39a | 5.3 |
| CG6959 | 10.1 | CG10943 | 6.8 | CG10345 | 5.3 |
| CG18157 | 10.1 | CG32372 | 6.8 | CG31780 | 5.3 |
| CG32718 | 10 | CG14007 | 6.8 | CG18477 | 5.3 |
| CG30411 | 10 | CG14500 | 6.8 | kappaTry | 5.3 |
| l(3)mbn | 10 | CG14014 | 6.8 | CG17816 | 5.2 |
| CG7298 | 9.9 | CG34113 | 6.8 | CG12490 | 5.2 |
| Or22a | 9.9 | CG41335 | 6.8 | CG12911 | 5.2 |
| CG30393 | 9.9 | elfless | 6.8 | CG10089 | 5.2 |
| CG16996 | 9.9 | CG16976 | 6.8 | CG10081 | 5.2 |
| CG6733 | 9.9 | CG18258 | 6.7 | CG17839 | 5.2 |
| CG34114 | 9.9 | CG9525 | 6.7 | CG5089 | 5.2 |
| CG34337 | 9.7 | CG31246 | 6.7 | CG17211 | 5.2 |
| CG32437 | 9.6 | CG31976 | 6.7 | CG3880 | 5.2 |
| Obp56d | 9.5 | CG34030 | 6.7 | prd | 5.2 |
| Cbp53E | 9.5 | CG14072 | 6.7 | CG16741 | 5.2 |
| Buffy | 9.5 | Hr4 | 6.7 | CG14974 | 5.2 |
| Eaat2 | 9.5 | Myo95E | 6.7 | CG31832 | 5.2 |
| mbl | 9.5 | CG13299 | 6.6 | CG33159 | 5.1 |
| Pdh | 9.4 | CG13457 | 6.6 | CG13228 | 5.1 |
| CG12910 | 9.4 | Cbl | 6.6 | Lcch3 | 5.1 |
| CG1076 | 9.4 | CG32970 | 6.6 | B4 | 5.1 |
| Tim17b2 | 9.4 | igl | 6.6 | CG40000 | 5.1 |
| CG17664 | 9.4 | hh | 6.6 | CG7271 | 5.1 |
| CG17086 | 9.4 | CG33225 | 6.6 | term | 5.1 |
| CG12960 | 9.3 | CG11778 | 6.6 | CG14076 | 5.1 |
| dve | 9.3 | CG31810 | 6.6 | CG14077 | 5.1 |
| CG14017 | 9.3 | CG12525 | 6.6 | CG12994 | 5 |
| Gr64f | 9.3 | CG4655 | 6.6 | Abd-B | 5 |
| robl22E | 9.2 | CG16815 | 6.5 | beat-VI | 5 |
| CG14294 | 9.1 | CG15534 | 6.5 | CG40040 | 5 |
| CG5160 | 9.1 | CG13569 | 6.5 | alpha-Est2 | 5 |
| CG17599 | 9 | CG3323 | 6.5 | CG32726 | 5 |
| CG9737 | 9 | Ets98B | 6.5 | CG13036 | 5 |
| Ance-5 | 9 | CG6184 | 6.5 | CG15227 | 5 |
| CG3669 | 9 | CG30154 | 6.4 | CG33523 | 5 |
| sick | 9 | CG5065 | 6.4 | TotC | 5 |
| CG8202 | 9 | Gr22a | 6.4 | CG31406 | 5 |
| B-H2 | 9 | CG12620 | 6.4 | CG13217 | 5 |
| CG15732 | 8.9 | CG30116 | 6.4 | CG14632 | 5 |
| CG14069 | 8.9 | CG15200 | 6.4 | CG34286 | 5 |
| CG30428 | 8.9 | per | 6.4 | CG32087 | 5 |
| CG18467 | 8.9 | CG15666 | 6.4 | CG15905 | 5 |
| CG4950 | 8.9 | Ilp7 | 6.3 | Sobp | 5 |
| CG5404 | 8.9 | Tsp66A | 6.3 | Blimp-1 | 5 |
| CG14608 | 8.9 | C901 | 6.3 | CG6663 | 5 |
| Adgf-E | 8.8 | mid | 6.3 | CG32694 | 5 |
| CG31788 | 8.8 | CG30364 | 6.3 | Cyp313a2 | 5 |
| Spz3 | 8.7 | CG30365 | 6.3 | Gr47a | 5 |
| CG34297 | 8.7 | CG10969 | 6.3 | CG31542 | 5 |
| sala | 8.7 | CG6123 | 6.3 | CG10178 | 5 |
| CG4783 | 8.7 | CG13215 | 6.3 | CG13720 | 5 |
| Arpc3A | 8.7 | CG7045 | 6.3 | Ku70 | 2.1 |
| tin | 8.6 | CG9466 | 6.3 | Mre11 | 1.5 |
| AR-2 | 8.6 | CG10734 | 6.3 |  |  |
| CG5687 | 8.6 | CG32082 | 6.2 |  |  |
| CG4386 | 8.6 | Cyp4ac1 | 6.2 |  |  |
| IM23 | 8.6 | Hr51 | 6.2 |  |  |
| CG12506 | 8.6 | CG14626 | 6.2 |  |  |

Fold change more than five times in gene expression and *ku70*, *mre11* are show in the table.
