# Supplementary material for: Drosophila UTX Coordinates with p53 to Regulate ku80 Expression in Response to DNA Damage
Source: PLoS One. 2013 Nov 12;8(11):e78652. doi: 10.1371/journal.pone.0078652 (PMC3827076; doi:10.1371/journal.pone.0078652)
Supplement: Text S1 — Only contains the legend for Figure S1 and is not intended for publication. (DOC) [file pone.0078652.s003.doc]

**Supplementary data**

**Figure S1.** qRT-PCR analysis to confirm the knockdown efficiency of *ku80* RNAi.
